# Zn^2**+**^ Binding Shifts the Conformational Ensemble of α‑Synuclein Monomers toward Accelerated Amyloid Formation

**DOI:** 10.1021/jacs.5c11056

**Published:** 2025-09-25

**Authors:** Emily J. Byrd, Benjamin Rowlinson, Joel A. Crossley, David J. Brockwell, James F. Ross, Sheena E. Radford, Frank Sobott

**Affiliations:** Astbury Centre for Structural Molecular Biology, School of Molecular and Cellular Biology, Faculty of Biological Sciences, 8748 University of Leeds, Leeds LS2 9JT, U.K.

## Abstract

Alpha-synuclein (αS) is an intrinsically disordered protein (IDP) that can self-assemble into amyloid fibrils, undergoing a transition from disordered monomers to ordered β-sheet-rich fibrils. The amyloid state of αS is implicated in various synucleinopathies, most notably Parkinson’s disease (PD), in which αS fibrils accumulate as insoluble Lewy body deposits. Colocalized with αS in Lewy bodies are elevated levels of metal ions including Zn^2+^. We find *in vitro* that Zn^2+^ accelerates aggregation of N-terminally acetylated αS, decreasing the *t*_50_ ca. 5-fold, as measured by thioflavin T (ThT) fluorescence. Strikingly, the extent of Zn^2+^ binding (native mass spectrometry; MS) and shifts of the monomeric αS conformational ensemble toward compaction, measured using ion mobility MS (IM-MS) at different αS:Zn^2+^ ratios, mirror precisely the accelerated aggregation kinetics. Chemical shift perturbations in Nuclear Magnetic Resonance (NMR) spectroscopy were investigated together with molecular dynamics (MD) to map the Zn^2+^ binding sites and subsequent effects on conformation under identical solution conditions to those used in IM-MS. Zn^2+^ is found to predominantly interact with negative residues in the C-terminal region of αS but also His50 in the N-terminal region. This promiscuity in interactions potentially guides compaction of the protein chain by bridging residues between the N- and C-terminal regions through Zn^2+^ ion co-ordination. This study provides insights into the early stages of amyloid assembly, correlating aggregation kinetics with structural compaction in monomeric αS and highlighting the capability of native IM-MS to resolve complex structural ensembles of a disordered protein.

## Graphical abstract

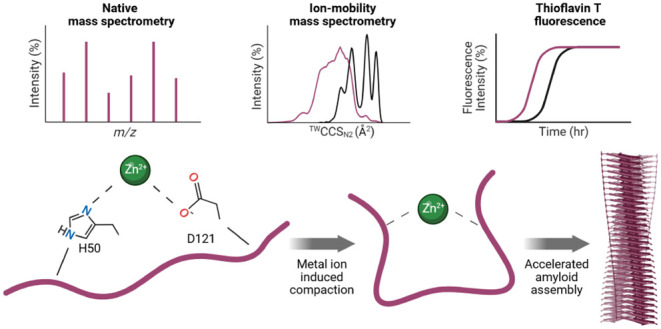

## Introduction

In its native, monomeric form, the protein alpha-synuclein (αS) is intrinsically disordered, adopting a broad conformational ensemble.^1–6^ αS, however, has a high amyloid propensity and can therefore undergo a major conformational transformation from unstructured to highly ordered β-sheet fibril architectures for which many polymorphs have now been solved using cryo-electron microscopy.^7–12^ Despite this extensive characterization of the fibril architecture, key mechanistic details of the transition from the disordered monomeric protein to the highly structured amyloid fibril remain largely unresolved. The ability to assemble amyloid fibrils associated with neurodegeneration is found in a range of proteins such as amyloid β, TAR DNA-binding protein 43 (TDP-43) and tau,^13–17^ which are implicated in various diseases such as Alzheimer’s disease and amyotrophic lateral sclerosis.^18–20^ Deposition of αS fibrils, in particular, is associated with Parkinson’s disease (PD) and other synucleinopathies.^21–25^ The pathological hallmark of PD is the accumulation of insoluble cellular deposits called Lewy bodies which contain amyloid fibrils composed of many forms of αS, including N-terminally acetylated, phosphorylated at S129, C-terminal truncations and N-terminal truncations of various sequence lengths.^26–35^ As well as the dense accumulation of proteins, Lewy bodies contain marked abundances of heavy metal ions such as Cu^2+^, Mn^2+^ and Zn^2+^ which correlate with elevated metal ion abundances detected locally in PD brain samples.^36^

Amyloid assembly is a complex process, and an array of oligomeric intermediates have been reported to form early in assembly, some of which are hypothesized to act as nuclei for fibril growth.^37–43^ Such heterogeneity poses challenges in characterizing the causative mechanisms of PD and the early stages in αS amyloid assembly,^13,44^ and has largely frustrated the targeting of early species for therapeutic intervention. Nonetheless, the very first stage of αS amyloid fibril formation begins with the physiological state of the protein, most commonly the monomer, although a tetrameric, helical form of αS has also been postulated as the initiating species.^37,45–48^ αS monomers are intrinsically disordered and populate an ensemble of partially compact and extended conformational families present in equilibrium with one another, established through nuclear magnetic resonance (NMR), molecular dynamics (MD), small-angle X-ray scattering (SAXS), cross-linking mass spectrometry and single-molecule Förster resonance energy transfer (FRET).^3,38,49–54^ In previous work, the occurrence of compact and extended conformational families of αS were observed using ion-mobility mass spectrometry (IM-MS) through determination of the rotationally averaged collision cross section area in the gas phase.^6,53,55–58^

The short 140 amino acid sequence of αS displays features which govern its conformational properties. First, the N-terminal region (residues 1–60) is overall positively charged, containing 11 lysine residues and six imperfect KTKEGV repeats which have been proposed to function as a lipid-binding region.^59^ Interactions between αS, lipids and membrane surfaces have been shown to induce α-helical structure in this region.^59,60^ *In vivo,* αS is predominantly acetylated at the N-terminus, which has been shown to influence its membrane-binding affinity through its increased helicity.^28,61–65^ The N-terminal region also contains several missense point mutations that correlate with autosomal dominant forms of PD including V15A, A30P/G, E46K, A53E/T/V, H50Q, G51D and E83Q.^22,24,46,66–70^ The familial variants A53T, E46K, H50Q and E83Q accelerate the rate of amyloid fibril formation of the αS sequence,^22,67,71^ whereas G51D and A30P slow the rate of amyloid assembly.^72,73^ The “non amyloid-β component” (NAC) comprising residues 61–95 is a hydrophobic, amyloidogenic region and crucial for β-sheet assembly which promotes amyloid fibril formation.^33,74–77^ The C-terminal region (residues 96–140) contains a highly negatively charged sequence with 15 aspartic/glutamic acid residues with a high propensity for metal-ion binding (such as Cu^2+^, Cu^+^, Mn^2+^, Co^2+^, Zn^2+^) and Ca^2+^.^78–83^ Additionally, the C-terminal sequence is commonly phosphorylated *in vivo* at residues Y125, S129, Y133 or Y135 and is proposed to regulate αS amyloid assembly.^21,84–89^

The conformational ensemble of αS is thought to be finely tuned by the charge distribution along the protein sequence. Changes both in the sequence itself and in the environment in which the protein is found can result in shifts in the population of specific conformations within the ensemble.^3,57,58,90^ Intracellular Zn^2+^ within the brain, at a concentration of ∼50–150 μM,^91,92^ is predominantly inaccessible due to sequestration into organelles by cytosolic metal-binding proteins.^92,93^ Neuronal damage, such as during oxidative stress, can result in Zn^2+^ release into the cytosol.^94^ Indeed, synaptic Zn^2+^ can transfer from presynaptic neurons through Ca^2+^ channels or via vesicles, leading to colocalization with αS in neuronal synapses where the Zn^2+^ concentration is estimated to be around 100 μM.^92,95^ Here, we study how Zn^2+^ binding, as a hallmark of αS-associated pathology, perturbs the conformational equilibrium of N-acetylated αS monomers *in vitro* and that promotes amyloid assembly.^56^ Zn^2+^ has been shown to influence the conformational dynamics of αS^55^ , as well as the rate of amyloid formation.^55^ Markedly elevated Zn^2+^ concentrations are found in the brains of PD patients, specifically in Lewy bodies,^94^ whereas Ca^2+^ binding is believed to be an important physiological role of αS.^96^

Here we report a study combining native MS, IM-MS conformational studies, thioflavin T (ThT) measurements of amyloid assembly kinetics, NMR and MD simulations of Zn^2+^ binding to αS under identical conditions to those in IM-MS to examine the correlation between transient structures of αS and amyloid formation. We show that compaction of the αS protein chain induced by Zn^2+^ directly correlates with the half-time (*t*_50_) of amyloid formation. Combined, the data demonstrate the power of using IM-MS with NMR and MD to decipher a mechanism wherein an intrinsically disordered protein is coerced into an amyloid-promoting conformation through interactions with Zn^2+^.

## Experimental Section

### Protein Expression and Purification

Competent BL21 DE3 *E. coli* cells expressing NatB acetylase were prepared as follows. BL21 DE3 (Agilent) cells were transformed with the pNatB plasmid (Addgene 53613), and a single colony was used to inoculate a starter culture of LB medium overnight at 37 °C, 200 rpm. The overnight culture was used to inoculate 500 mL LB containing 25 μg/mL chloramphenicol until an OD_600_ of 0.6 was reached. Cells were pelleted at 4500*g* for 5 min. Cells were resuspended in 30 mM potassium acetate, 10 mM CaCl_2_, 50 mM MnCl_2_, 100 mM RbCl, 15% (v/v) glycerol, pH 5.8. Cells were incubated on ice for 5 min before pelleting and further resuspension in 10 mM MOPS, 75 mM CaCl_2_, 10 mM RbCl, 15% (v/v) glycerol, pH 6.5. Competent cells were stored at −80 °C until used.

Competent NatB-BL21 DE3 cells were transformed with a pET23a plasmid encoding wild type human full length αS to express both NatB and αS for N-terminal acetylation. Cells were grown in LB media containing 25 μg/mL chloramphenicol and 100 μg/mL carbenicillin until an OD_600_ of 0.6 was reached and protein expression was induced with the addition of 0.01 mg/mL isopropyl β-d-1-thiogalactopyranoside (IPTG) for 4 h at 37 °C, 200 rpm. Expressed protein was purified by cell lysis in 25 mM Tris-HCl pH 8.0, 100 μg/mL lysozyme, 50 μg/mL phenylmethylsulfonyl fluoride, 1 mM benzamidine and 20 μg/mL DNase and homogenized using an IKA T 18 ULTRA-TURRAX homogenizer (IKA, Staufen, Germany). The lysate was heated to 80 °C for 10 min and then centrifuged at 35000*g* for 30 min, 4 °C followed by ammonium sulfate precipitation (50% w/v) at 4 °C. The pellet containing αS was diluted in 20 mM Tris-HCl pH 8.0 and purified by anion exchange chromatography using a 350 mL Q-Sepharose fast flow anion-exchange column on an ÄKTA Prime (Cytiva, UK). Bound αS was eluted in a gradient of 0–500 mM NaCl, in 20 mM Tris-HCl pH 8.0 over a volume of 700 mL. Fractions containing αS were dialyzed against 5 × 5 L of 50 mM ammonium bicarbonate (3500 MWCO) at 4 °C and lyophilized. Freeze-dried protein was resuspended in 50 mM ammonium bicarbonate at 5 mg/mL and loaded onto a HiLoad 26/60 Superdex-75 column for size exclusion chromatography eluted in 50 mM ammonium bicarbonate. Fractions containing αS were pooled and lyophilized.

### Kinetics of Amyloid Formation

Kinetics of αS amyloid formation were monitored in a 96-well, nonbinding, flat-bottom, half area microplate (Corning, USA; 10438082) containing one Teflon polyball (1/8” diameter; Polysciences Europe, Eppelheim, Germany) per each well of sample. Samples of 100 μL volume containing 100 μM αS with 20 μM Thioflavin T in 20 mM ammonium acetate, pH 7.5 were incubated at 37 °C shaking at 600 rpm in a FLUOstar omega plate reader (BMG Labtech, Ortenburg, Germany). Fluorescence intensity was measured by exciting fluorescence at 440 ± 10 nm and collecting emission at 482 ± 12 nm using a bandpass filter. Zinc acetate (Sigma-Aldrich, 1724703) was added at concentrations of 100 μM, 300 μM, 500 μM, 750 μM, 1.5 mM, 2.5 mM or 4 mM. Results were blank corrected using wells containing 20 μM ThT in 20 mM ammonium acetate, pH 7.5 and Zn^2+^ but lacking protein and the results then normalized to the maximum fluorescence value of each curve. The time in which half of the maximum ThT fluorescence was reached was calculated using AmyloFit.^97^

### Negative Stain Transmission Electron Microscopy (TEM)

Five μL of sample from the ThT plate at the end point of each reaction was loaded onto a glow discharged (30 s, Pelco Easi-glow), 400 mesh continuous carbon grid, and incubated for 2 min. The sample was blotted and washed twice with H_2_O before being blotted staining twice with 1% (w/v) uranyl acetate. Grids were imaged on a Tecnai F20 electron microscope (FEI) T12 with a Ceta CCD detector in the Astbury Electron Microscopy Facility (University of Leeds), using a nominal magnification of 23300×.

### Native Ion Mobility-Mass Spectrometry (IM-MS)

Native IM-MS experiments were performed on a Synapt G1 HD mass spectrometer (Waters, Wilmslow, UK) with traveling (T-wave) ion mobility and a nano-ESI (nESI) source using *in-house* generated gold and palladium coated capillaries. Zn^2+^ titrations were acquired using 20 μM N-acetylated αS, in 20 mM ammonium acetate pH 7.5, with 2 μM, 10 μM, 20 μM, 40 μM, 60 μM, 100 μM, 150 μM, 200 μM, 300 μM, 400 μM, 500 μM or 800 μM zinc acetate each in triplicate. Protein was desalted using a ZebaSpin desalting column 7k MWCO (0.5 mL; Fisher Scientific). Instrument parameters were set at: capillary voltage 1.4 kV, source temperature 30 °C, sampling cone 18 V, extraction cone 1.0 V, trap collision energy 5.0 V, transfer collision energy 2.0 V, trap DC bias 30 V, IM wave velocity 300 m/s, IM wave height 7.0 V. Gas pressures in the instrument were: trap cell 0.0256 mbar, IM cell 0.36 mbar. The IM cell was calibrated according to the Bush database^98^ using denatured cytochrome c (charge states 13+ to 19+), myoglobin (charge states 15+ to 24+) and ubiquitin (charge states 7+ to 13+) at 10 μM in 50% (v/v) acetonitrile, 0.1% (v/v) formic acid.

Using MassLynx 4.1 (Waters, Wilmslow, UK), % binding was calculated by extracting peak areas of bound spectral peaks versus the peak area of the unbound spectral peak, % compaction was calculating by extracting the integrated peak area of compact conformations (4.0–7.0 ms) versus extended conformations (7.1–10 ms) via arrival time distributions of the 8+ charge state which is the most conformationally diverse and represents both compact and extended structures of αS.^53,55,56,99^ All measurements were taken in triplicate and the average of three replicates was plotted.

### *K*_d_ Fitting with UniDec Data Collector

Zn^2+^ titrations were acquired using 20 μM αS in 20 mM ammonium acetate pH 7.5, with 0, 2, 10, 20, 40, 60, 100, 150, 200, 300, 400, 500, or 800 μM of zinc acetate. Mass spectrometry was performed on an Orbitrap Eclipse (Thermo Fisher Scientific, CA, USA) and raw data was extracted using FreeStyle (Thermo Fisher Scientific). Instrument parameters were as follows. Positive ion spray voltage: 1400 V, ion transfer tube: 275 °C, *m*/*z* range: 200–4000, detector type: Orbitrap, RF Lens: 150%), Normalized AGC target: 100%, microscans: 5. *K*_d_ values were obtained by fitting titration curves in the data collector utility from UniDec with the following parameters: “what to extract: Raw data”, “How to extract: Local max”, “Window: 2 Th”, “Number of proteins: 1”, “Number of ligands: 5 (based on significant binding above a minimum signal/noise threshold of 3)”, “Protein and Ligand Models: All KD’s free” enabling normalization of data and extraction.^100^

### (^1^H, ^15^N)-HSQC NMR Spectroscopy

^15^N labeled N-terminally acetylated αS was expressed in 0.5 L of minimal medium containing 3.75 g Na_2_HPO_4_, 5 g K_2_HPO_4_, 4.5 g K_2_SO_4_, 5 g KH_2_PO_4_, 0.5 g ^15^NH_4_Cl supplemented with 1 mM MgCl_2_, 100 μM CaCl_2_ and 0.8% (w/v) glucose and purified identically to unlabeled protein. 2D (^1^H, ^15^N) HSQC spectra of 100 μM αS, with 1, 2.5, 5, 10, 15, and 25 mol equiv of zinc acetate in 20 mM ammonium acetate, pH 7.5, were recorded at 25 °C using a Bruker AVANCE III 750 MHz spectrometer equipped with a triple resonance TCI-cryoprobe. Spectra were recorded with 2048 and 512 complex points in the F2 and F1 dimensions, respectively. Spectral widths were set to 14.1 ppm centered on 4.7 and 30.0 ppm centered on 119 ppm for F2 and F1 dimensions, respectively. Spectra were processed using TopSpin 4.0.6 and analyzed using CCPN analysis software.^101^ Chemical shifts were calculated using eq 1.

1 $$\mathrm{CSP}=\sqrt{{\left(\Delta \delta_{H}\right)}^{2}+0.14{\left(\Delta \delta_{N}\right)}^{2}}$$

### MD Simulations

Molecular dynamics simulations were performed to investigate cation-residue binding preferences, bridging, persistence, and exchange. Three monomeric starting conformations of αS were extracted from PDB structures 2N0A, 8A9L, and 8ADS. Missing terminal residues in 8A9L and 8ADS were added from 2N0A using PyMOL [The PyMOL Molecular Graphics System, Version 2.0, Schrödinger, LLC]. For each starting conformation, three simulations were conducted with 5 ions of Zn^2+^. The N-terminus was acetylated, and charge neutralization was achieved with Na^+^ and acetate ions. AMBER Molecular dynamic input files were generated using AmberTools,^102^ using tleap and parmed, with protonation states set for pH 7.0. Each system was solvated in a truncated octahedron with OPC (Optimal Point Charge) water,^103^ with a 14 Å gap between the protein and the periodic boundary. Force field parameters from leaprc.protein.ff19SB, leaprc.water.OPC, and frcmod.ionslm_1264_opc^104^ were applied, with modifications for hydrogen mass repartitioning and the 12–6–4 point ions made in parmed. The systems were minimized using the steepest descent algorithm, equilibrated at 303.15 K with backbone restraints for 5000 steps, and simulated using 4 fs time steps in AMBER22 on the ARC4 HPC at the University of Leeds. Trajectories were generated for 1.5 μs, with coordinates saved every 500 ps for analysis. Initial postprocessing was carried out with cpptraj and RMSD matrix calculations and ion-protein distance measurements were performed in PyMOL. Data processing and visualization were performed using bash and Python, utilizing numpy, pandas, seaborn, matplotlib, pylab, and mpl_toolkits.

## Results

### Global Compaction of αS Determined by IM-MS Correlates with the Rate of Amyloid Formation

Using native MS, Zn^2+^ binding to N-acetylated αS was monitored at different concentrations of added zinc acetate (0, 2, 10, 20, 40, 60, 100, 150, 200, 300, 400, 500, or 800 μM; ratios of zero to 40-fold molar excess) to 20 μM αS in ammonium acetate buffer. Zn^2+^ bound to all αS charge states ranging from 5+ to 17+ with equal apparent affinity, determined by the comparable relative intensities (Zn^2+^ bound/Zn^2+^ free) of ligand-bound states. In native MS, charge states reflect multiple protonation events (or attachment of an equivalent number of metal ions) according to the solvent accessible surface area of a protein’s conformation; here lower αS charge states (5–8+) correspond to more compact conformations and higher charge states (9–17+) originate from more expanded structures.^105,106^ Example native mass spectra in Figure 1 show a shift in the charge state distribution toward more compact conformations (panels on the left-hand side) as well as identifying the number of Zn^2+^ binding events to the 8+ charge state with increasing excess of the metal ion (panels on the right-hand side).

**Figure 1:**
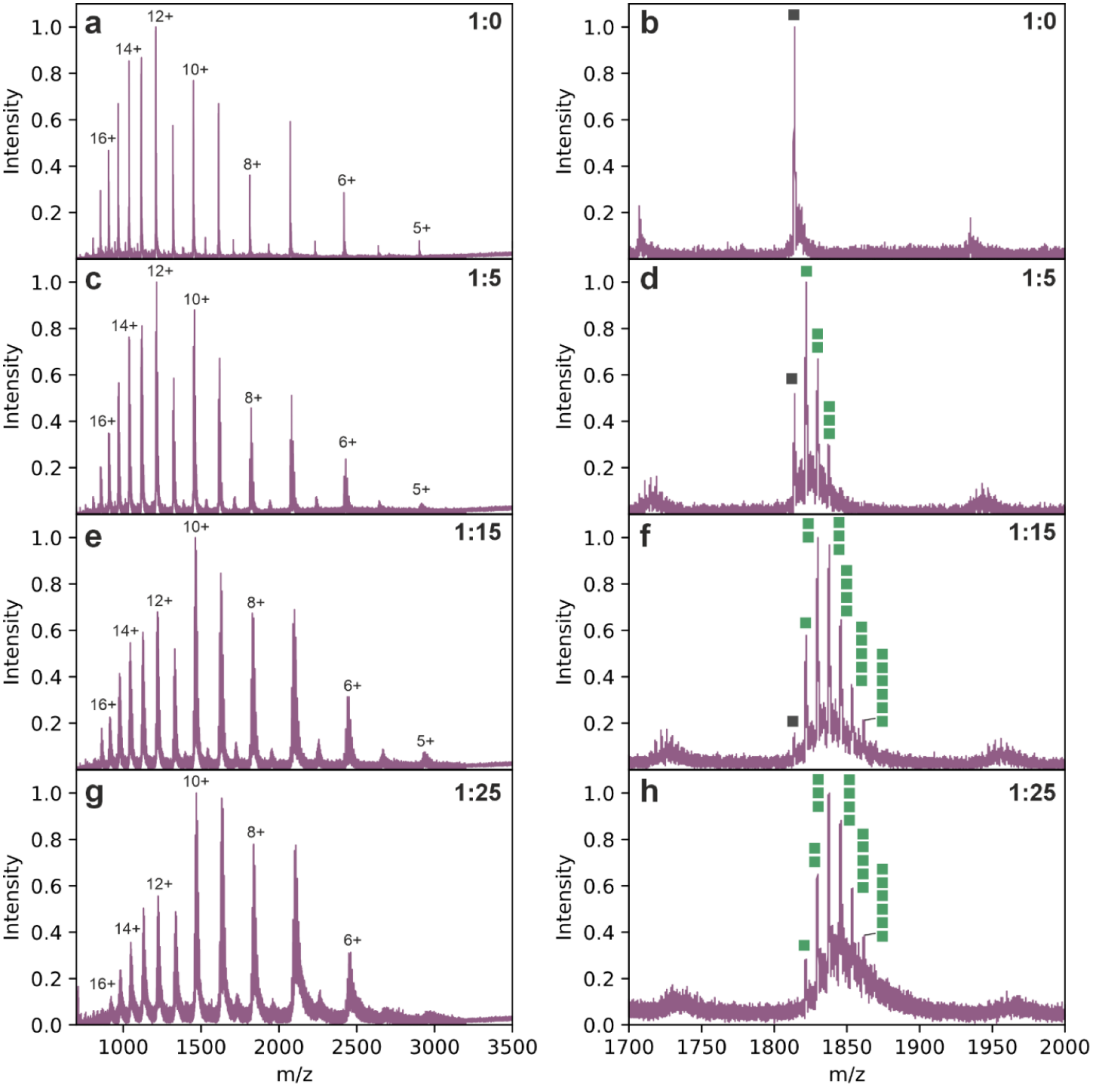
Native mass spectra identify Zn^2+^ binding to αS. Native mass spectra in the left panel represent the full native nESI charge state distribution (5+ to 17+) with increasing excess of Zn^2+^, and native mass spectra on the right panel show zoomed spectra of the 8+ charge state where the apo state is shown by a gray square and Zn^2+^ binding events are shown as green squares. Spectra are shown for the molar ratios (a and b) 1:0, (c and d) 1:5, (e and f) 1:15 and (g and h) 1:25 (αS: Zn^2+^ added).

The effect of Zn^2+^ on the amyloid assembly kinetics of αS was investigated next using a Zn^2+^ titration measured by ThT fluorescence under identical solution conditions to those used in native MS experiments (Figure 2). Figure 2b shows that Zn^2+^ has a dramatic effect on the rate of amyloid assembly, reducing the time taken to reach half of the maximum fluorescence (*t*_50_) up to 5-fold at a 1:15 molar ratio of αS to Zn^2+^. In order to compare the effects of Zn^2+^ binding on the *t*_50_ of amyloid formation with the conformational properties of the monomer, we used native nESI with IM-MS under the same conditions with Zn^2+^ titrated at molar ratios from 1:1 to 1:40 (αS:Zn^2+^). We assessed the extent of Zn^2+^ binding (percent occupancy) for each step of this titration by summing the integrated areas of the 8+ charge state peaks with between one and six Zn^2+^ ions bound, relative to the apo form, measured using native nESI MS (Figures 1 and 2e, red curve). Next, IM-MS was used to determine the effect of Zn^2+^ binding on the conformational ensemble of αS monomers via their rotationally averaged collision cross section (CCS).^107,108^ IM-MS provides a precise measurement of the rotationally averaged size of each ligand bound state.^58,109^ The IM-MS fingerprint of the αS ensemble clearly shows a global trend toward compaction in the presence of Zn^2+^, both by an increase in the abundance of low charge states and also as a shift within the individual ion mobility profiles (Figure 3).

**Figure 2:**
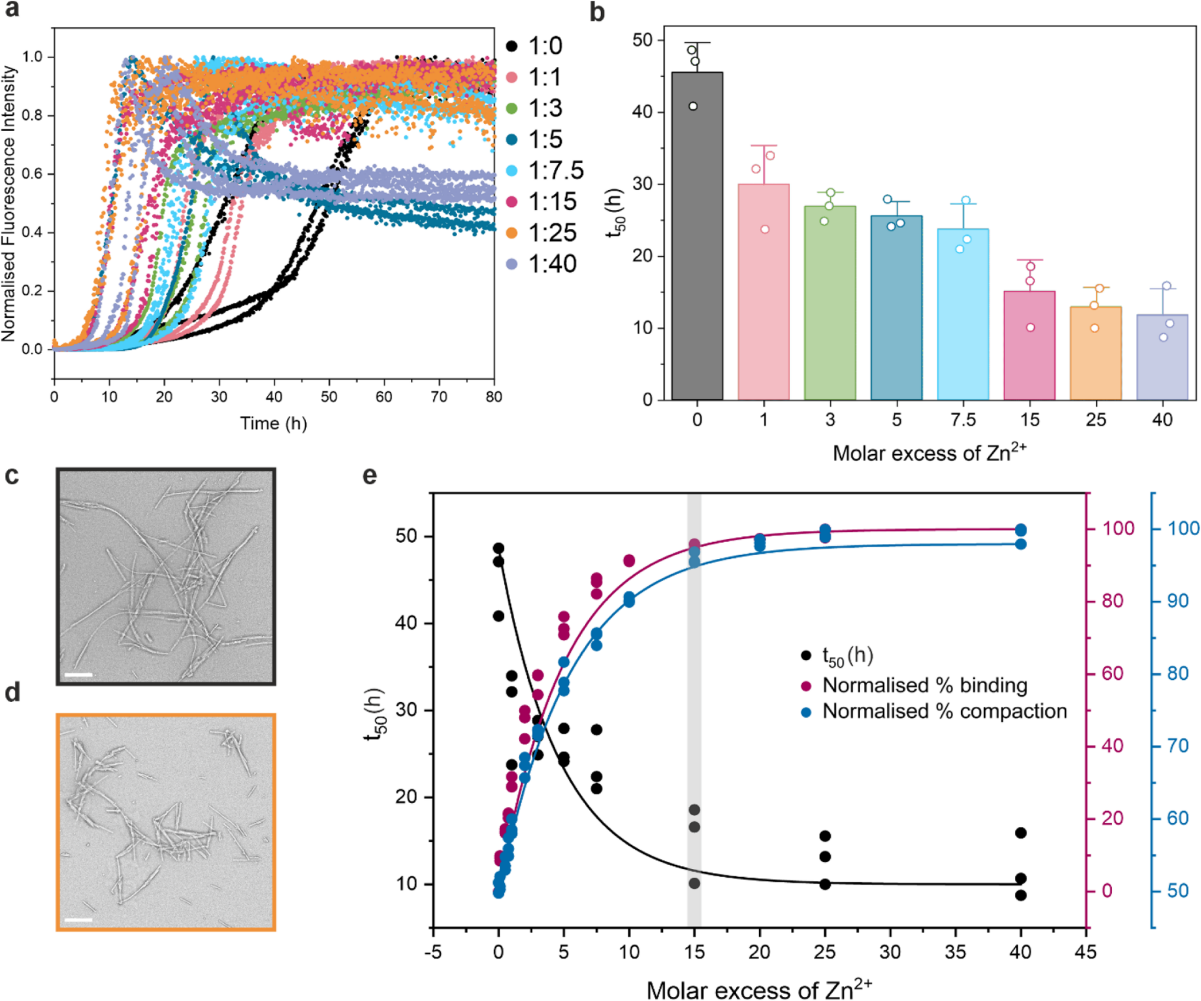
Correlating ion mobility with *t*_50_ directly links compaction with amyloid aggregation propensity in the presence of Zn^2+^. (a) The molar excess of Zn^2+^ was increased stepwise in the presence of N-acetylated αS with amyloid assembly measured by ThT fluorescence. The molar ratio (αS:Zn^2+^) is shown alongside in the key. (b) A plot of the reduction of the t_50_ of amyloid assembly of αS by Zn^2+^ from three replicate values for each molar excess of Zn^2+^. Values for *t*_50_ were calculated using AmyloFit.^97^ Negative stain TEM of (c) αS amyloid fibrils in the absence of Zn^2+^ and (d) αS amyloid fibrils with Zn^2+^ added at a 25-fold molar excess. The scale bar corresponds to 200 nm. (e) The effect of Zn^2+^ concentration on the kinetics (t_50_; black) of amyloid formation, overall compaction (ion mobility; blue) of the 8+ charge state and percent occupancy by binding (native MS; red) to the 8+ charge state saturates at around a 15-fold molar excess of Zn^2+^ (gray bar).

**Figure 3:**
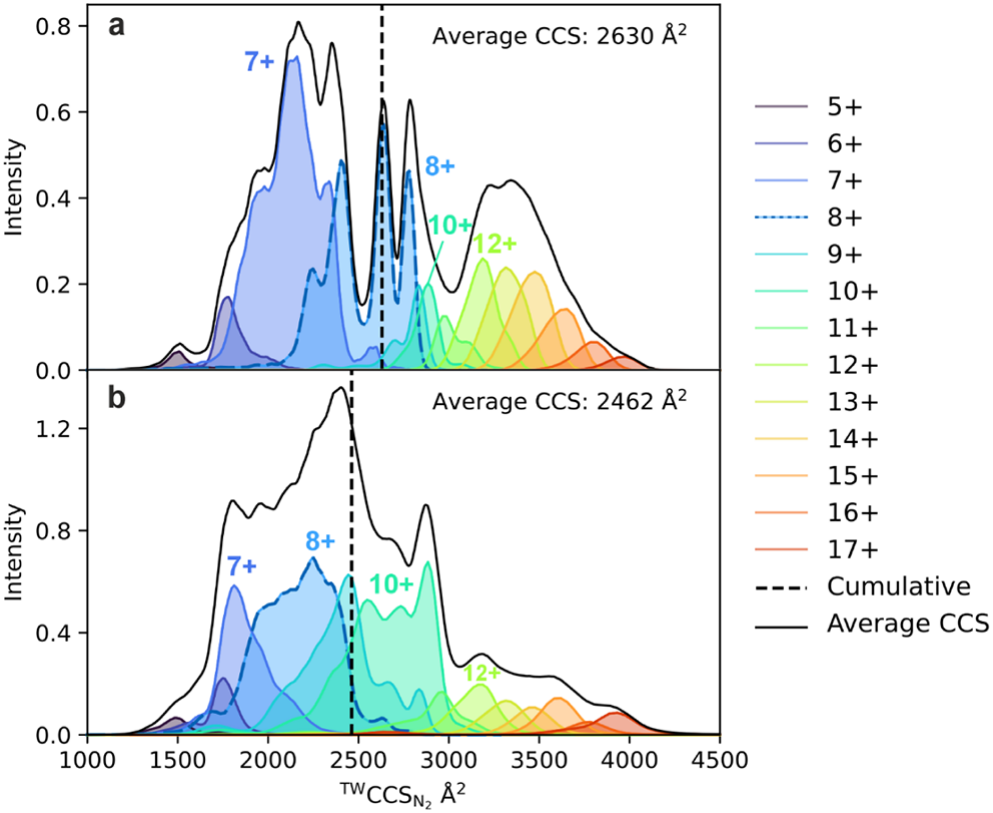
Compaction of αS ^TW^CCS_N2_ using IM-MS. The full IM-MS fingerprint of N-acetylated αS in the absence of Zn^2+^ (a) and in the presence of a 25-fold molar excess of Zn^2+^ (b). Each color distribution represents the IM-MS fingerprint for the respective charge state (including Zn^2+^ bound states in panel b) identified by native MS as shown by the key to the right-hand side, the 8+ charge state is shown in light blue with a dashed blue line. The black solid line represents the sum of all charge states and the vertical black dashed line identifies the average CCS value.

Similarly to the percentage of Zn^2+^ binding, we derived a measure for compaction from the complex traveling wave ion mobility data in nitrogen gas (^TW^CCS_N2_; Figures 3 and S1). Strikingly, the percent occupancy of binding and the shift in ion mobility toward compact species at different αS:Zn^2+^ ratios (Figure 2e, blue curve) mirrors precisely the *t*_50_ values measured using ThT fluorescence (Figure 2e, black curve). Saturation of binding, compaction and the effect of decreasing *t*_50_ all level off around a 15-fold molar excess of Zn^2+^ (Figure 2e). These different types of experimental data suggest a direct correlation between compaction of the monomeric conformation of αS with its amyloidogenicity.

### Zn^2+^ Binding to αS Occurs Stepwise

Binding of Zn^2+^ to αS follows a hyperbolic trend (Figure 2e) which suggests there are several equivalent binding sites, and that stepwise ion binding occurs until saturation. Native MS is a powerful method for reporting the stoichiometry of ligand-binding events to different charge states and/or conformations and can also be used to provide semiquantitative binding information based on peak intensities.^110–113^ Assuming that peak intensities in native mass spectra reflect binding in solution, where ideally all species present ionize with the same efficiency,^114^ the affinity of a ligand to individual charge states at each binding step (stoichiometry) can be determined.^115^ Since nESI charging depends on solvent accessible surface area (SASA),^105,106^ binding affinities can be determined and compared for different conformational families, demonstrated here through the analysis of individual charge states and their conformational profiles determined by CCS calibration (Figure 3). Binding affinities are calculated by fitting the relative intensities of each of the bound peaks (αS bound to one Zn^2+^ up to five Zn^2+^) versus unbound peaks as a function of increasing ligand concentration and this therefore requires high resolution data which resolves each bound state for accurate extraction of peak areas.^116^

Native nESI mass spectra of Zn^2+^ binding to αS used for fitting apparent *K*_d_ values are shown in Figure S2. Generally, by first deconvolving the spectra, accurate peak areas can be extracted for each ligand-bound state and subsequent *K*_d_ fitting can be performed (see Experimental Section). Using the data collector node in UniDec to fit *K*_d_, apparent binding affinities were determined for αS with Zn^2+^ without deconvolving spectra in order to analyze multiple charge states representing conformational families^100,117^ and are shown in Figure 4. For the first binding event, *K*_d_ values for the compact 7+ charge state, (58 μM), the conformationally highly heterogeneous 8+ ions (54 μM) and the extended 10+ (55 μM) and 12+ ions (27 μM) are similar, with slightly weaker binding observed for the 12+ charge state, suggesting that Zn^2+^ binds to all conformational families with comparable affinity and stoichiometry (similar number of binding events shown in the native spectra in Figure S2). These findings contrast with the binding of small drug-like molecules such as epigallocatechin gallate (EGCG) and dopamine to αS which were found to be selective by charge state, and therefore conformation, and also shift the charge state distribution toward more compact and more extended states, respectively.^118^ Here, each subsequent Zn^2+^ binding affinity was similar ruling out cooperative binding, and suggesting instead stepwise, independent binding events.

**Figure 4:**
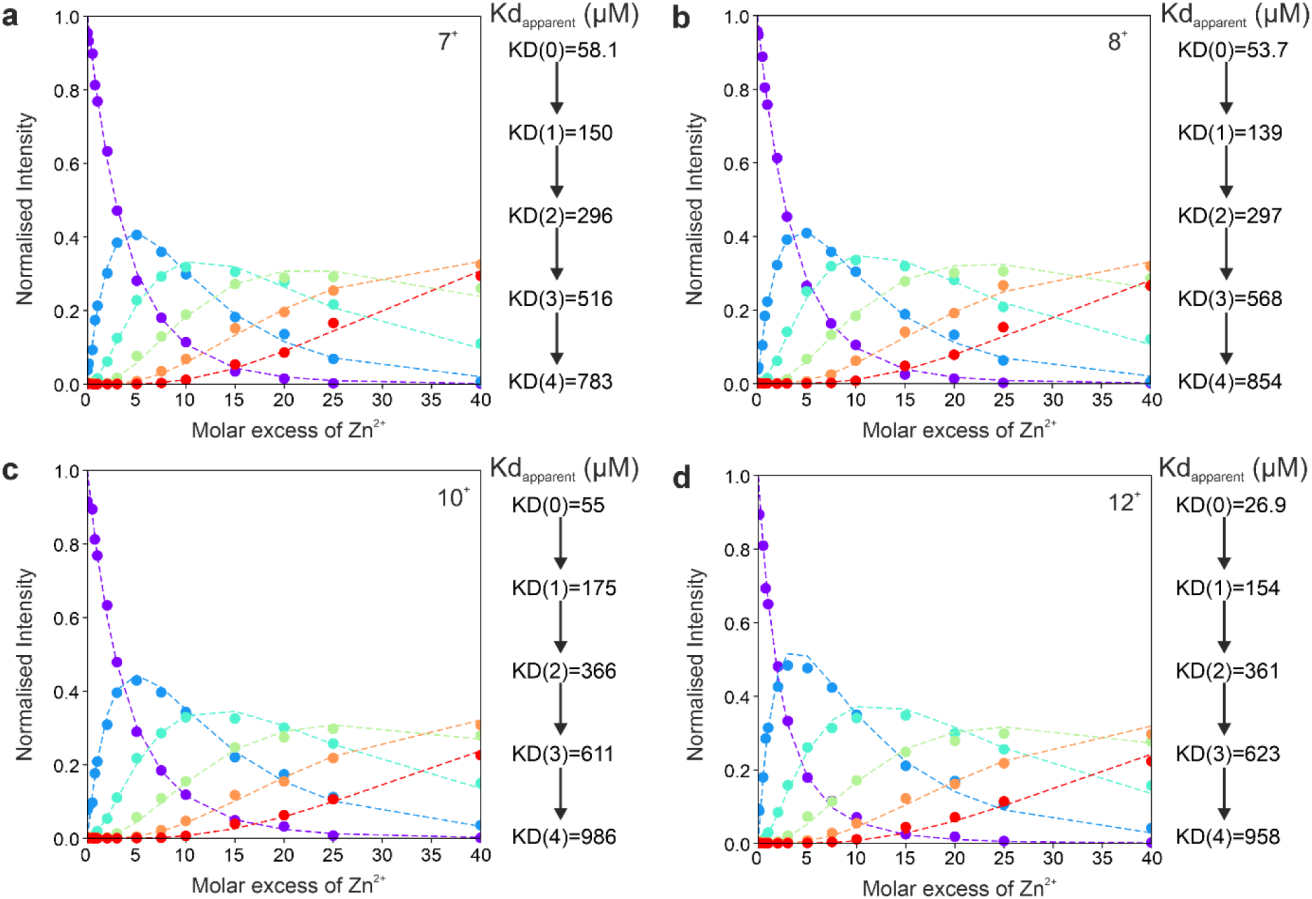
Determination of apparent*K*_d_values of Zn^2+^ binding by native MS. (a) *K*_d_ fitting for the 7+ charge state showing the unbound species (purple), 1 bound (blue), 2 bound (cyan), 3 bound (green), 4 bound (orange) and 5 Zn^2+^ bound (red), *K*_d_ value determinations are shown on the right. *K*_d_ fitting for the 8+ (b), 10+ (c) and 12+ (d) charge state. Data were fitted using the data collector node in UniDec and accounted for the peak areas for the apo up to the five Zn^2+^ bound peak in the multistate binding model.^100^ nESI mass spectra are shown in Figure S2.

### Different Residues in αS Bind to Zn^2+^ by NMR Analysis

Previous studies by NMR have identified the specific residues and regions of unacetylated αS that interact with different divalent metal ions, Ca^2+^ was found to bind preferentially to C-terminal residues containing carboxyl groups and Cu^2+^ to both the C-terminus and H50 in the N-terminal region.^79,80,82,83,94,115–119^ Zn^2+^ has also been observed to interact with C-terminal residues as well as residues in close proximity to H50.^79,80,82,83,96,119–123^ To determine the location(s) of Zn^2+^ binding sites to N-acetylated αS under conditions matching our IM-MS and ThT experiments (20 mM ammonium acetate, pH 7.5), ^1^H–^15^N-HSQC spectra were obtained of ^15^N-labeled αS in increasing concentrations of Zn^2+^ (Figure 6).

The results show that with increasing concentrations of Zn^2+^, a gradual shift in peak position was observed for some residues consistent with rapid chemical exchange between the Zn^2+^ bound and unbound states (Figure 5). In agreement with previous studies,^80,83,124^ the most significant chemical shift changes identified H50 and D121 as key residues for Zn^2+^ binding (Figure 5b–e). We further noticed that peaks with significant chemical shifts reduced in intensity with increased Zn^2+^ concentration (Figure S3), suggesting that Zn^2+^ binding occurs on an intermediate time scale by NMR, although the relatively small change in intensity demonstrates that Zn^2+^ binding is dominated by a fast exchange regime. Chemical shift perturbations (CSPs) are sensitive to changes in the local chemical environment of each of the backbone amide bonds, and hence they report on direct binding effects as well as changes due to possible structural rearrangements. The magnitude of CSPs is shown in Figure 5 and, in agreement with literature observations, the greatest CSPs were observed for H50 and D121, as well as Y125 and S129.^83,119^

**Figure 5:**
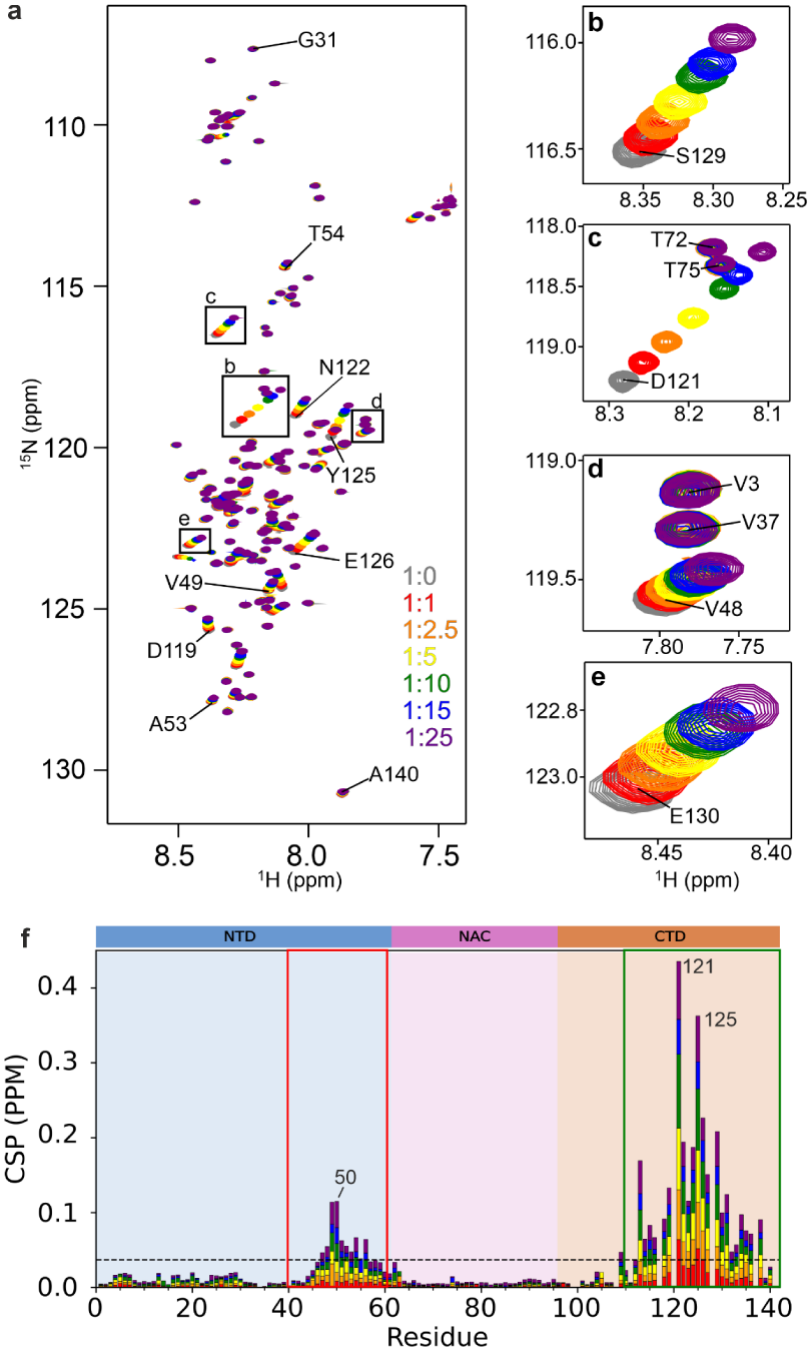
2D (^1^H, ^15^N)-HSQC NMR spectra showing a Zn^2+^ titration with αS. (a) The concentrations of Zn^2+^ used were 0 μM (gray), 100 μM (red), 250 μM (orange), 500 μM (yellow), 1 mM (green), 1.5 mM (blue) and 2.5 mM (purple) which resulted in the molar ratios presented in the right-hand key. The panels on the right-hand side show zoomed regions of the spectra identifying cross-peaks of residues (b) S129, (c) D121 which shows significant movements in peak positions, (d) V48 along with V3 and V37 and (e) E130 Spectra were acquired at 25 °C in 20 mM ammonium acetate, pH 7.5, 100 μM N-acetylated αS. (f) CSPs after the addition of Zn^2+^ at 25 °C in 20 mM ammonium acetate, pH 7.5. The dashed line represents the mean CSP value for all residues of 0.0063. Significant shifts are identified for residues 48–52 in the N-terminal domain (red box) and in the C-terminal region (residues 113–138) in agreement with previous literature (green box).^125^

### Zn^2+^ Induced Compaction as Observed by Molecular Dynamics Simulations

To further explore the structural basis of Zn^2+^ induced compaction of αS, molecular dynamics (MD) simulations were carried out using N-terminally acetylated αS starting from three different experimentally determined structures (PDB: 2N0A, 8A9L, and 8ADS). Each simulation was initiated from a monomer; initially a conformation found in a fibril structure but which rapidly relaxed to form a dynamic ensemble characteristic of an IDP within 0.5 ns of the simulation. Each simulation was run for 2.0 μs to capture the earliest time scales of metal ion interaction and resultant conformational rearrangement (Figure 6). The simulations support a model where Zn^2+^ ions retained a dense hydration shell which remained largely intact over 2 μs (Figure S4d) and have limited direct contact with the protein backbone or side chains of αS (Figure 6a), reducing the effective free Zn^2+^ coordination sites available for high-affinity binding. This is consistent with the weak apparent *K*_d_ values derived from native MS (∼50 μM; Figure 4), and the absence of cooperative binding.

**Figure 6:**
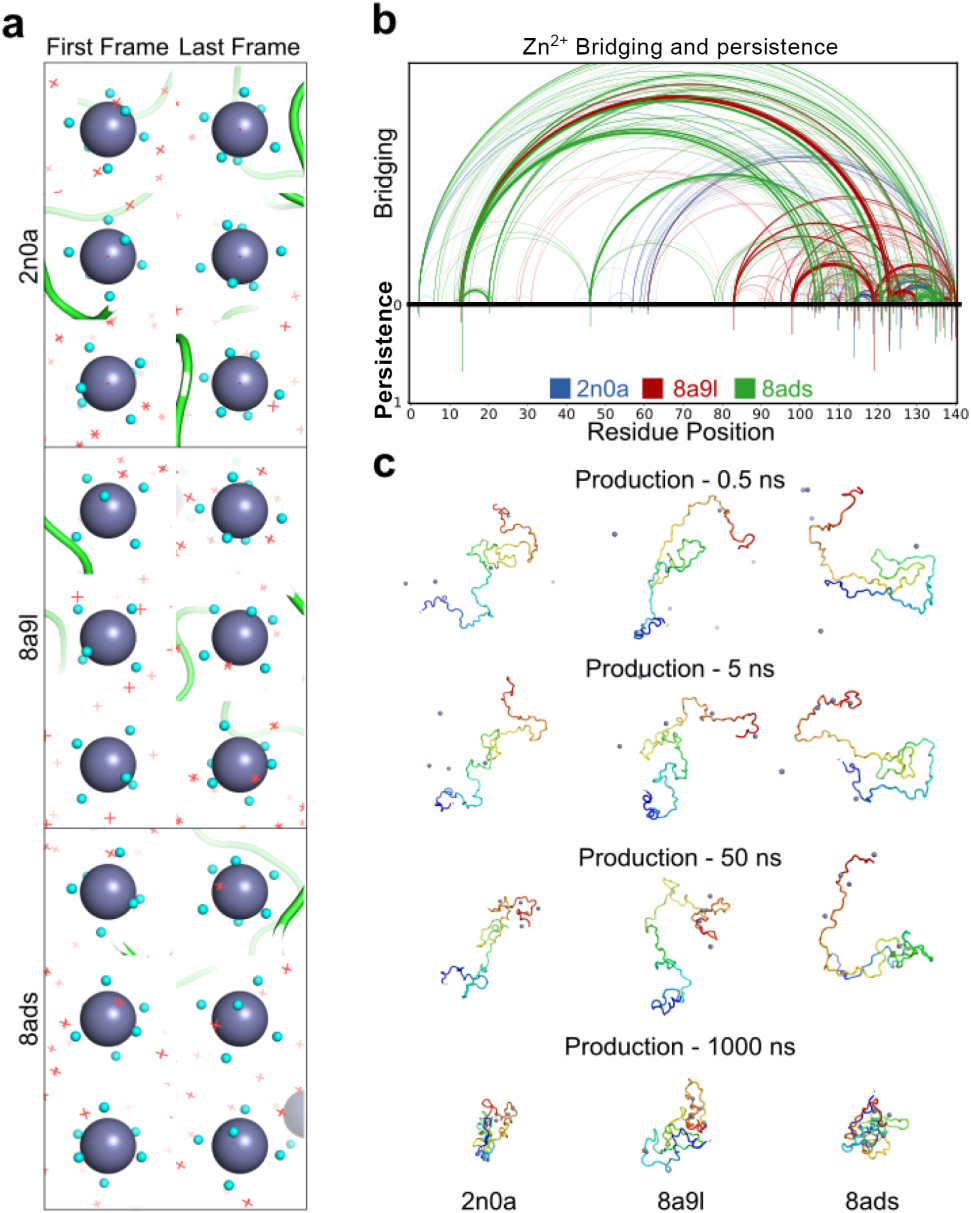
Molecular dynamics simulations of N-terminally acetylated αS monomers with Zn^2+^reveal transient N-to-C terminal bridging and compaction. (a) Snapshots from three examples of the first and last frames of MD simulations (2n0a, 8a9l, 8ads PDB starting models), showing initial hydration waters (cyan spheres) coordinating to Zn^2+^ ions (larger purple spheres). At the end of the simulation (2 μs) the same (initial) water molecules are still bound, these reduce the Zn^2+^–αS coordination propensity at these time scales. New water molecules are shown as red crosses. (b) Arc diagram of Zn^2+^ bridging interactions between residue pairs along the αS sequence. Bridges occur between N-terminal and C-terminal residues but are transient as shown by short persistence times (the scale from 0 to 1 indicates the proportion of bound states compared to unbound states across the length of the simulations) between a residue and any Zn^2+^ ion, below axis). (c) Time-resolved MD snapshots at 0.5, 5, 50, and 1000 ns. Zn^2+^ ion binding and resultant compaction of αS is observed over time.

Zn^2+^ interacted via longer-range electrostatic interactions (∼4.5 Å) with clusters of acidic residues in the C-terminal region and formed bridged interactions with residues in the N-terminal region (Figure 6b). These interactions were observed across all three starting conformers, though the exact residue pairings varied. Importantly, these long-range interactions were observed to be dynamic, with lower contact times to specific αS residues compared to other typically studied ions (such as Ca^2+^ as previously reported,^126^ and in Figure S4), reinforcing a model where Zn^2+^ exerts its effects on conformation and amyloid assembly of αS through dynamic, multivalent and weakly specific electrostatic interactions rather than through fixed structural coordination bonds.

Over the course of the simulations, αS adopted increasing compaction of the protein chain (Figure 6c). This was evident from bridging of the αS N- and C-termini showing the protein chain collapsing. Although Zn^2+^ interactions remained locally short-lived, they were retained in the αS collapsed form by electrostatic interactions and the result was a noticeable shift toward more collapsed conformational families, consistent with the conformational compaction observed by IM-MS. As the simulation progressed, three (of a total of 82) hydration cage waters were displaced by D120, E14 and E124 carboxyl groups, at times 0.5, 1.2, and 1.6 μs, displaying a slow but gradual exchange of hydration cage waters with αS residues (Figure 6a). These results support a model in which weak but multivalent and transient Zn^2+^ binding events promote dynamic chain rearrangements on the μs time scale that result in an increase in overall compaction of the protein upon eventual Zn^2+^ hydration cage displacement. This is a marked difference to the behavior of Ca^2+^ binding, which forms immediate coordination with carboxylate groups and individually persist for over 2 μs, Ca^2+^ binding is believed to be a physiological function of αS (see Figure S4).^127^

## Discussion

In this study, we demonstrate that Zn^2+^ binding to monomeric, N-acetylated αS results in characteristic shifts in its conformational ensemble toward more compact states. The extent of this structural rearrangement shows excellent correlation with acceleration of the rate of amyloid assembly. In the case of PD, Zn^2+^ levels are observed to be elevated in affected brain regions, particularly in association with Lewy bodies.^36,124,128–130^ While Zn^2+^ interactions with αS have been studied previously, the mechanistic basis by which Zn^2+^ influences the early stages of αS amyloid assembly has remained unclear.^79,80,124^

Here, we used native nESI-MS and IM-MS to show that Zn^2+^ binding to αS influences its conformational behavior and its amyloidogenicity, which draws on the power of IM-MS to separate and individually track different charge states and conformational families.^6,57,131,132^ We found a direct correspondence between the extent of Zn^2+^ binding to αS and overall compaction measured using IM-MS, which also correlates with the reduction of t_50_ of amyloid formation (acceleration of αS amyloid assembly) by ThT fluorescence. Other metal ions have previously been shown to accelerate αS aggregation including Mn^2+68^,^129^ and Cu^2+68^,^133^ Indeed, the greatest effects on amyloid assembly and compaction of αS have been identified for the addition of Zn­(CH_3_CO_2_)_2_ and therefore hence Zn^2+^ was chosen here to represent pathological metal ion binding.^130^ These findings also highlight the ability of careful and sensitive ionization by native nESI-MS to capture diverse conformational states of an intrinsically disordered protein from solution for gas-phase analysis by ion mobility. The addition of Zn^2+^ leads to a reduction in the t_50_ of αS, with an apparent *K*_d_ of ∼50 μM per binding event consistent with biologically relevant concentrations of Zn^2+^ which range from low nM to the high μM range (100 μM).^134^ The observed affinities, while only being semiquantitative, show that binding is not a cooperative process, but instead occurs in a stepwise manner to available sites on the protein chain.

While these interactions are undoubtedly driven by electrostatics, physiological Ca^2+^ and pathological Zn^2+^ show clear differences in their binding behavior. A key advantage of the native IM-MS approach is the ability to simultaneously resolve individual metal ion-bound states and their associated conformational profiles, thus linking stepwise binding with apparent affinities while also providing information on whether there are conformational preferences or effects on conformation upon Zn^2+^ binding. With *K*_d_ values of around 50 μM, Zn^2+^ binds weakly to αS across charge states from 7+ to 12+, with the more extended 12+ conformations showing ca. 2× weaker binding. These findings are reinforced by data in Figure S2 where the comparable affinity of Zn^2+^ binding suggests little or no conformational selectivity of binding (i.e., across the charge state spectrum). On the other hand, once bound, Zn^2+^ coordination results in significant and characteristic conformational effects toward more compact states that occur predominantly on the lower charge states (below 12+, Figure 3) of αS. The data presented here support this model whereby compact conformational families become more compact due to metal ion co-ordination and collapse of the densely negatively charged C-terminal region. This was further illustrated by MD simulations (Figure 6). By contrast, more extended conformational families remain extended upon binding. This suggests that the most amyloid-prone conformational family in the presence of Zn^2+^ is the most compact, and that this conformation constitutes only a small proportion of the overall ensemble of αS but plays a key role in driving amyloid assembly. A more detailed analysis of the complex fingerprints obtained by IM-MS (Figure 3) is beyond the current capabilities of computational modeling.

Different metal ions have been shown to interact with different regions of αS.^79,80,82,83,96,119,120,121,122,123^ Cu^2+^ ions were found to have a high propensity to interact with the N-terminal residues and H50, while Ca^2+^ preferentially interacts with the C-terminal region.^96^ Similarly, Zn^2+^ is predicted to preferentially bind to H50 and the C-terminal region, particularly 119DPDNEA124, preferring carboxyl groups over nitrogen containing groups.^118^ The interaction of Cu^+^ with N-terminally acetylated αS has also been investigated. Similarly to nonacetylated αS, key binding sites for Cu^+^ were observed in the N-terminal region (M1 and M5), with the addition of the N-terminal acetyl group also increasing the occurrence of α-helical structural content at the N-terminus.^122^ Interestingly, we show here and previously,^55,126^ that Zn^2+^ enhances the amyloidogenic behavior of αS and compacts monomers more readily than Ca^2+^.^55,126^ To explore this further, we conducted MD simulations comparing Zn^2+^ and Ca^2+^ binding to N-acetylated αS under matched conditions within the first 1000 ns to observe the initial events of binding. These findings revealed striking differences where Ca^2+^ binding is dominated by persistent, long-lived interactions that firmly attach Ca^2+^ ions to specific carboxylate-rich residues in the C-terminal region, displacing water molecules and forming stable coordination networks (Figure S4). In contrast, Zn^2+^ binding is much more dynamic and transient and the hydration shell surrounding Zn^2+^ is more tightly retained (Figure 6a). (^1^H, ^15^N)-HSQC NMR chemical shift perturbations at H50 (N-terminus) and D121 (C-terminus), indicate two independent, low-affinity Zn^2+^ binding sites with fast exchange (Figure 5). These NMR-mapped binding sites and resultant chemical shifts complement our MD findings and suggest remote structural effects may also contribute. These weak but promiscuous interactions are consistent with native nESI-MS findings in Figure 5 that show stepwise, noncooperative Zn^2+^ binding across multiple charge states, with apparent *K*_d_ values in the ∼50 μM range. In simulations, Zn^2+^ ions bridge the N-terminal region with C-terminal acidic residues. These contacts exhibit short persistence, underscoring a high degree of flexibility. Despite this initial weak binding, MD shows that αS progressively compacts over time with a slow increase in Zn^2+^ coordination-based interactions (Figure 6c), supporting our experimental IM-MS data showing increased populations of compact conformational families.

In our IM-MS, native MS, ThT fluorescence and NMR experiments we used a wide range of Zn^2+^ concentrations up to a large molar excess in the mM range (with differences due to method-specific dynamic ranges) in order to study molecular effects well into saturation of Zn^2+^ binding. To enable cross-technique comparison we therefore analyze responses as a function of αS:Zn^2+^ equivalents and fractional occupancy. Importantly, the key behaviors that we observe-stepwise, noncooperative binding, compaction, and reduced *t*_50_, all emerge in the overlapping submillimolar regime and plateau at ca. 15-fold excess of Zn^2+^ per αS. Thus, our mechanistic conclusions are based on trends with ligand equivalents rather than any single concentration point. ThT fluorescence detects amyloid fibrils, and a substantial number must be produced for detectable signal, which underlies the lag phase. For example, fibrils can be present in the lag phase but not detected.^135^ ThT fluorescence would not detect oligomers, especially small numbers which do not produce a signal.^40,136^ MS, by contrast can detect low levels of oligomers, but not fibrils. Hence the two methods are not in contradiction, but synergistic, each providing different information.

Taken together, the results presented provide a molecular explanation for how a seemingly weak and transient interaction, such as electrostatic effects driven by Zn^2+^ ions, can exert a significant influence on the amyloid-assembly kinetics of an IDP. By transiently bringing together the N- and C-termini, Zn^2+^ may expose the NAC domain, effectively priming the protein for amyloid assembly. The difference in interaction strength and dynamics between Zn^2+^ and Ca^2+^ could also explain why Zn^2+^ leads to faster amyloid assembly as well as different preferential binding loci. This highlights the functional relevance of metal ion coordination flexibility in modulating IDP behavior, and suggests that weak, multivalent binding can act as an effective trigger for conformational reorganization and amyloid assembly, as shown here combining IM-MS and MD simulations.

## Conclusion

Our work highlights the power of using native nESI-MS and IM-MS to resolve the conformational heterogeneity of IDPs in response to environmental perturbations. By combining native nESI-MS, IM-MS, ThT fluorescence, NMR and MD simulations under aligned buffer conditions, we revealed how subtle shifts in conformational populations, driven by transient metal ion interactions, can have pronounced effects on amyloid propensity. This integrative approach provides a robust framework for studying early events in amyloid assembly.

## Supplementary Material



## Notes

IM-MS plots of the 8+ charge state; native mass spectra for *K*_d_ fitting; (^1^H, ^15^N)-HSQC NMR intensity changes; MD of αS with Ca^2+^ (PDF)
