# Supplementary material for: Zn2+ Binding Shifts the Conformational Ensemble of α‑Synuclein Monomers toward Accelerated Amyloid Formation
Source: J Am Chem Soc. 2025 Sep 25;147(40):36464–77. doi: 10.1021/jacs.5c11056 (PMC12512189; doi:10.1021/jacs.5c11056)
Supplement: Supplementary file 1 [file ja5c11056_si_001.pdf]

Supporting information

Zn^{2+} Binding Shifts the Conformational Ensemble of α-Synuclein Monomers Toward Accelerated Amyloid Formation

Emily J. Byrd, Benjamin Rowlinson, Joel A. Crossley, David J. Brockwell, James F. Ross*, Sheena E. Radford*, Frank Sobott*

Astbury Centre for Structural Molecular Biology, School of Molecular and Cellular Biology, Faculty of Biological Sciences, University of Leeds, Leeds, LS2 9JT, UK

* To whom correspondence should be addressed:
f.sobott@leeds.ac.uk, s.e.radford@leeds.ac.uk and j.f.ross@leeds.ac.uk

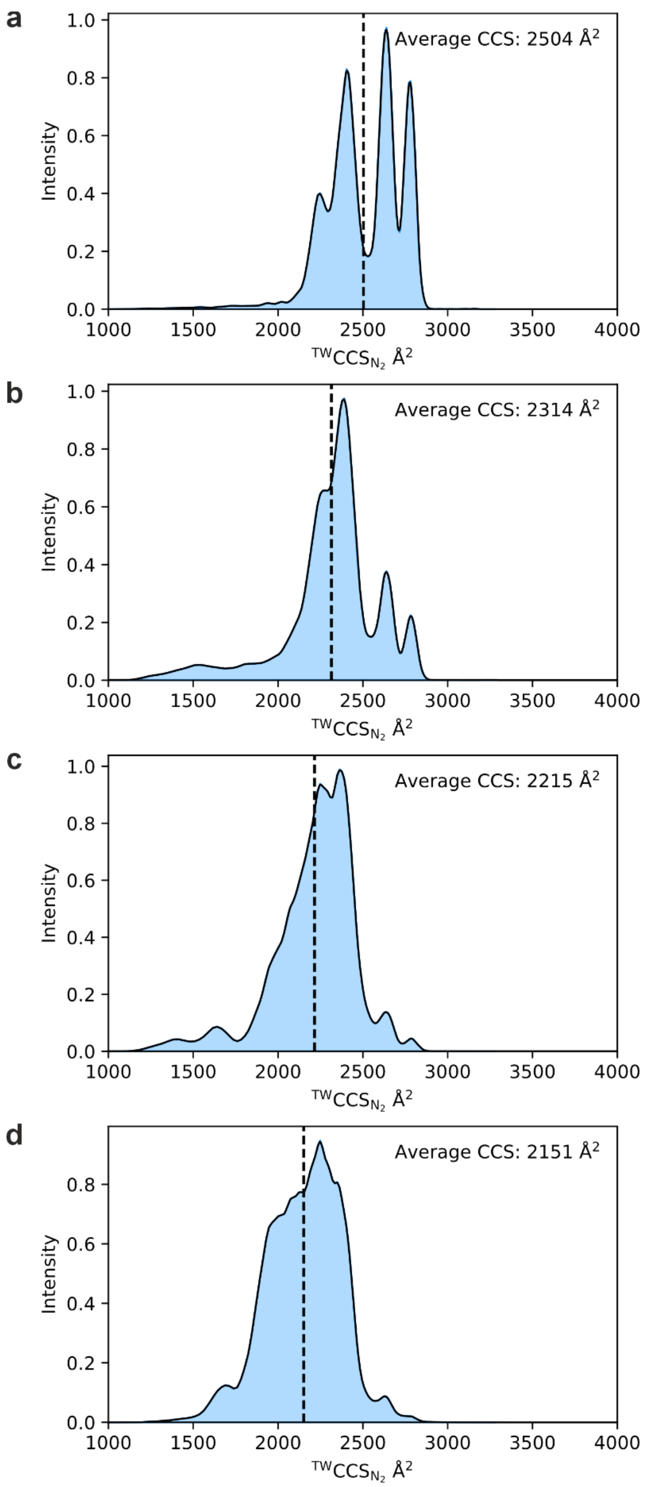

Figure S1 Compaction of the 8+ charge state of αS by $^{TW}CCS_{N2}$ using IM-MS. *The IM-MS fingerprint of the 8+ charge state in the absence of Zn^{2+} (a) and in the presence of a 5-fold (b), 15-fold (c) and 25-fold molar excess of Zn^{2+} (d). The black solid line represents the cumulative fit of all charge states and the black dotted line identifies the average CCS value.*

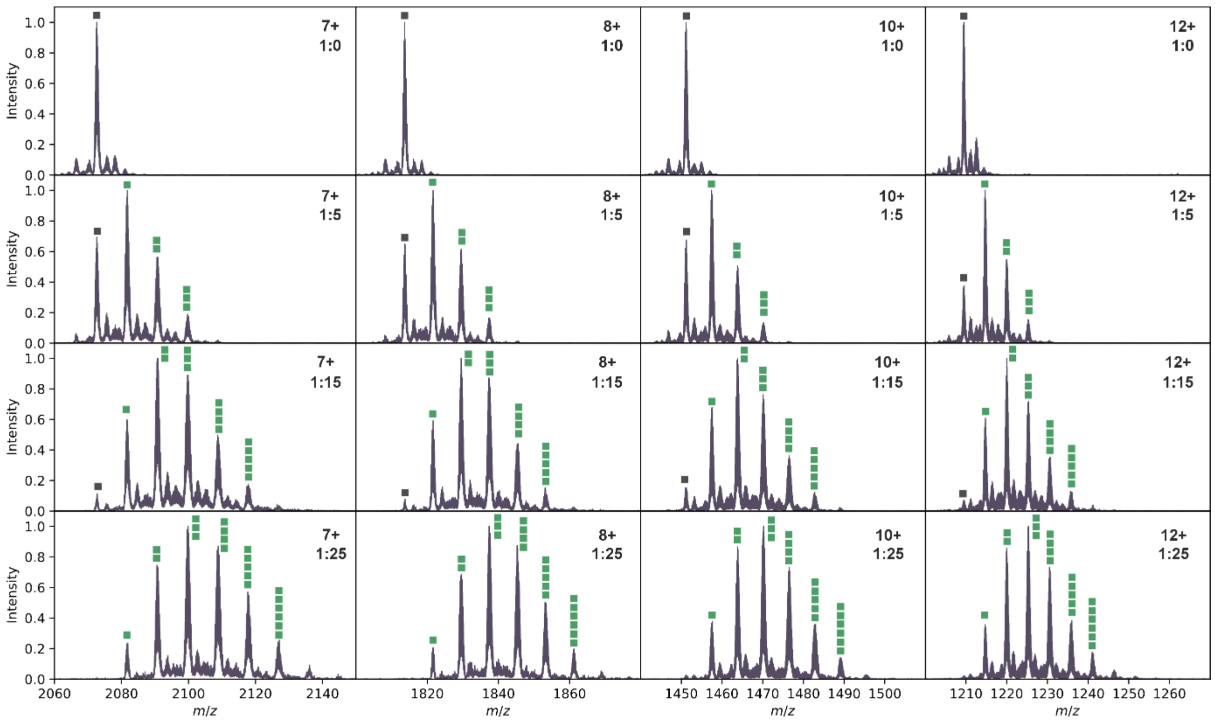

Figure S2 Native MS of Zn^{2+} binding affinity and stoichiometry with αS for K_d fitting. *Native mass spectra for the 7+, 8+, 10+ and 12+ charge states of αS used for K_d fitting are shown for the protein:zinc acetate molar ratios of 1:0, 1:5, 1:15 and 1:25. The unbound state of αS is denoted by a grey square and subsequent Zn^{2+} bound states are labelled with green squares.*

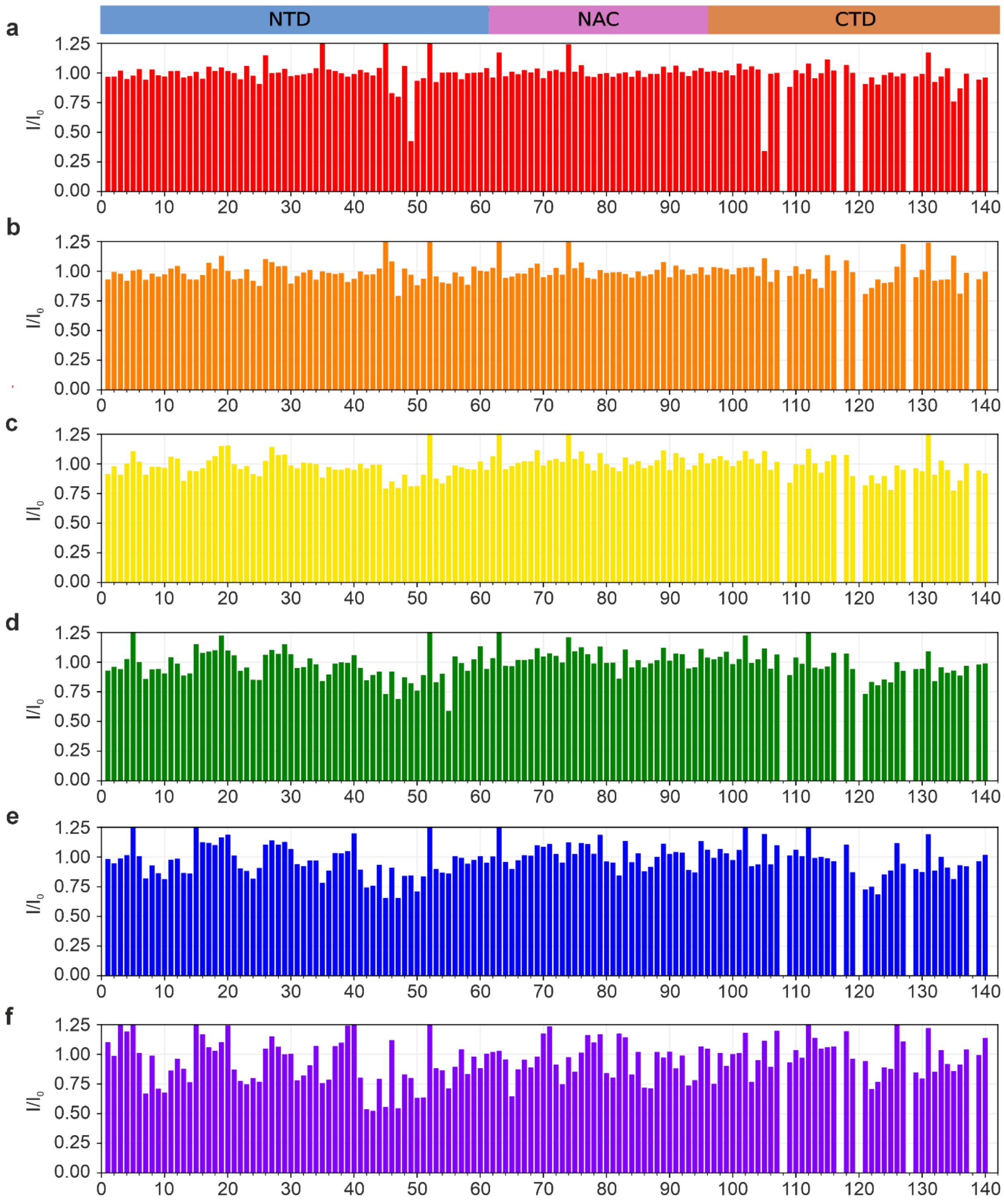

Figure S3 Intensity change expressed as a ratio of the (^1H, ^{15}N)-HSQC NMR resonances of N-terminally acetylated αS in the presence of (I) or the absence of (I_0) Zn^{2+}. *The concentrations of Zn^{2+} were (a) 100 μM; red, (b) 250 μM; orange, (c) 500 μM; yellow, (d) 1 mM; green, (e) 1.5 mM; blue and (f) 2.5 mM; purple, the concentration of αS was 100 μM. Residues with zero intensity are missing assignments.*

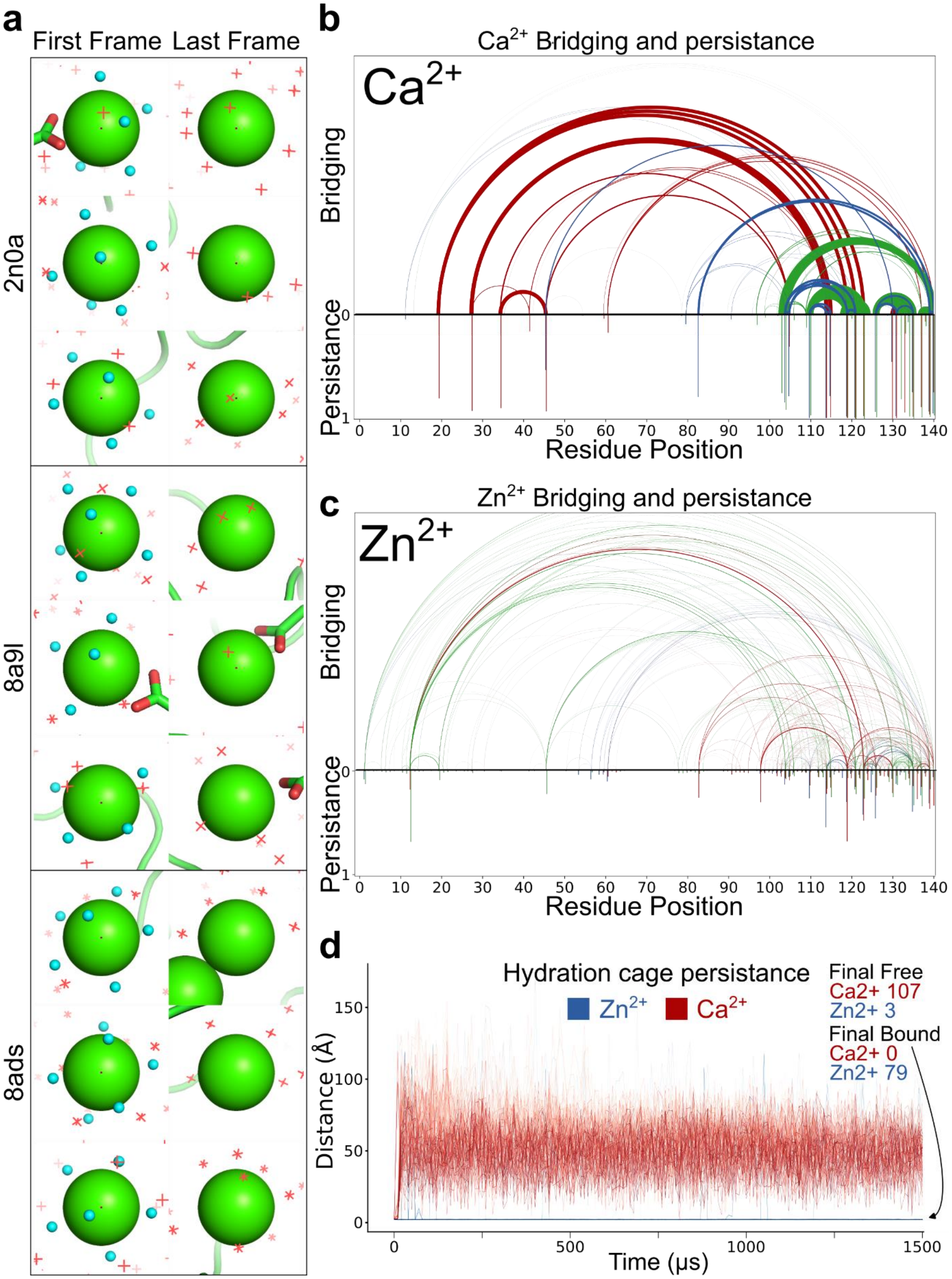

Figure S4. Molecular dynamics simulations of αS with Ca^{2+} reveal persistent N-to-C terminal bridging and compaction. *(a) Snapshots from the first and last frames of MD simulations (2n0a, 8a9l, 8ads PDB starting models), showing hydration shells (cyan spheres) surrounding Ca^{2+} ions (green) consist of replaceable new water molecules (red crosses), which strengthens Ca^{2+}-αS*

coordination. (b) Arc diagram of Ca^{2+} bridging interactions between residue pairs along the αS sequence. Bridges occur between N-terminal and C-terminal residues but are transient as shown by short persistence times (below axis; the scale from 0 to 1 indicates the proportion of bound states compared to unbound states across the length of the simulations). (c) as Figure 6b, but with the bridging widths normalised to Ca^{2+} bridging above (b) to allow direct comparison. (d) distance of initial hydration cage waters from initially bound ions over time. Zn^{2+} hydration waters remain bound over the simulation, Ca^{2+} hydration waters dissociate quickly.
